# Spleen autotransplantation on liver versus greater omentum for simultaneous injuries of liver and spleen: an experimental study

**DOI:** 10.1097/MS9.0000000000002198

**Published:** 2024-05-20

**Authors:** Mohammad Eslamian, Erfan Sheikhbahaei, Behnam Sanei, Alireza Rahmati, Masoud Moslehi, Hamidreza Zefreh, Arshin Ghaedi, Alireza Firouzfar

**Affiliations:** a Department of Surgery; b Department of Medical Physics, School of Medicine, Isfahan University of Medical Sciences, Isfahan; c Trauma Research Center, Shahid Rajaee (Emtiaz) Trauma Hospital; d Student Research Committee, School of Medicine, Shiraz University of Medical Sciences, Shiraz, Iran

**Keywords:** autotransplantation, liver, spleen, splenectomy, trauma

## Abstract

**Purpose:**

In a simulated situation of simultaneous spleen and liver trauma, we aimed to compare the outcomes of treating both injuries with spleen autotransplantation on the omentum (SAO) alongside hepatorrhaphy versus spleen autotransplantation as a patch on the liver parenchyma.

**Methods:**

A total of 24 rats were separated into two groups: the SAO and the spleen autotransplantation on the liver. They underwent a uniform and simultaneous procedure involving full-thickness injuries to the left lobe of the liver and grade 4 spleen injuries. We measured hemoglobin, white blood cell (WBC), complement (C3 and C4), and immunoglobulin G, M, and A (IgG, IgM, IgA) levels before and 4 weeks after the surgery. We utilized Technetium-99m scintigraphy to evaluate the posttransplant splenic graft functions 4 weeks after the surgery.

**Results:**

The two groups had no significant difference in the hematologic and immunologic factors before surgery. However, both procedures significantly reduced hemoglobin, C3, IgG, and IgA levels (all *P*<0.05). WBC counts significantly increased in the SAO group, whereas the IgM level decreased after the intervention (*P*<0.05). WBC was increased in the SAO group, while IgM and IgA were decreased in the SAO group. The Technetium uptake was similar between the two groups (*P*=0.3).

**Conclusions:**

In simultaneous spleen and liver injuries, the autotransplantation of splenic into the liver parenchyma appears to be a promising surgical approach for preserving spleen function and hepatorrhaphy at the same time instead of doing them separately.

## Introduction

Highlights

- Autotransplanting splenic tissues as a patch maintains splenic function.
- Patching the liver with the spleen may be a useful procedure to preserve splenic function.
- With a spleen patch, there is no need to use hemostat agents to control hepatic bleeding.

About 1% of blunt abdominal traumas have simultaneous liver and spleen injuries, of which 30% of them need surgical attention^1^. The management of liver and spleen dual trauma is contingent upon the extent of the injuries and the patient’s overall health status^2^. No operative management and minimally invasive approaches are currently the recommended standard of care for stable patients experiencing blunt abdominal trauma^2^. However, the only therapeutic choice for unstable patients or highly damaged splenic injuries (such as in burst hilum) is splenectomy^3^, and in order to avoid overwhelming postsplenectomy infections, spleen preserving methods have been evolved^4,5^. One of the many spleen-preserving techniques is autotransplantation, in which spleen fragments are put into different body parts^5^. Autotransplantation of the spleen is a controversial procedure, yet it is the only way to maintain splenic function in the event of severe trauma^4,6^. Spleen autotransplantation has been performed on animals since 1986 and on humans since 2004–2005^7^. The effectiveness of spleen autotransplantation in different areas in both humans and experimental animals has been proven^1,8,9^. Where to place the spleen tissue is one of the subjects that have been discussed in the literature^10^. Transplanting pieces of spleen parenchyma into pouches of the greater omentum is one of the most common methods of spleen autotransplantation^6^. This method is popular due to its beneficial portal drainage and revascularization^7^. However, this procedure has several risks, including chronic anemia, intestinal obstruction, and necrosis of the implants^8,9^. The liver and spleen are subcostal parenchymal organs rich in blood supply and have similar functions in the reticuloendothelial system^10^.

Due to the mentioned complications and similarities between the liver and spleen, hypothetically, the splenic tissue can be transplanted into the liver in simultaneous liver and spleen trauma where hepatorrhaphy and splenectomy are inevitable^8^. This opportunity has been investigated in limited studies, and despite their promising findings and bright future potential, they found controversial results, which necessitate further evaluation^8,11,12^. Therefore, in the present experimental study, the outcomes of two methods of splenic autotransplantations in simultaneous spleen and liver laceration compared: spleen autotransplantation by insertion of spleen fragments in lacerated liver parenchyma versus traditional spleen autotransplantation in the omentum (SAO).

## Materials and methods

### Animals

The work is prepared and reported per the criteria of the ARRIVE guideline^13^. Based on the previous reports on this field, 24 rats weighing between 290 and 350 g and 6–8 weeks old were used in this study. The body weight distribution of rats was the same in both groups (*P*=0.1). No surgical or medical interventions were recorded among any of the rats. The animals were kept in standard cages, maintained in a controlled environment free from pathogens, with a constant ambient temperature of 24°C and humidity with unrestricted access to water and food. The rats underwent a fasting period prior to the surgical procedure. The rats were equally divided into two groups: SAO group (*n*=12) and spleen autotransplantation on the liver (SAL) group (*n*=12), utilizing a block randomization technique that ensured matched subjects within each block with no blinding or further controlling for confounders. The exclusion criteria encompassed rats that expired during the study due to causes unrelated to the investigation.

### Intervention

Splenic autotransplantation by insertion of spleen fragments in the liver parenchyma in a simultaneous spleen and liver laceration requiring splenectomy and hepatorrhaphy as the intervention and autotransplantation of splenic fragments in omentum with hepatorrhaphy as control was performed on each group. One surgeon performed the procedure for all subjects under the same standard conditions. Each rat was anesthetized with 50 mg/kg pentobarbital sodium intraperitoneally and placed supine on a surgery table with the assistance of an experienced veterinarian. The abdomen skin was then shaved via hair removal cream (Veet, French) and disinfected with 10% betadine. In both groups, we used a no. 15 scalpel and a sterile environment to make a 5-cm midline incision on the abdomen.

Based on the size of the spleen, we simulated a grade 4 splenic injury (from the cortex to the hilum of the spleen) to harmonize the trauma for all of the rats; then, after traumatization and total separation of the spleen, the splenocolic ligament, short gastric vessels, and the splenic artery and vein were double-ligated with silk sutures (4-0 Taft, Yazd, Iran) and cut.

**Figure 1:**
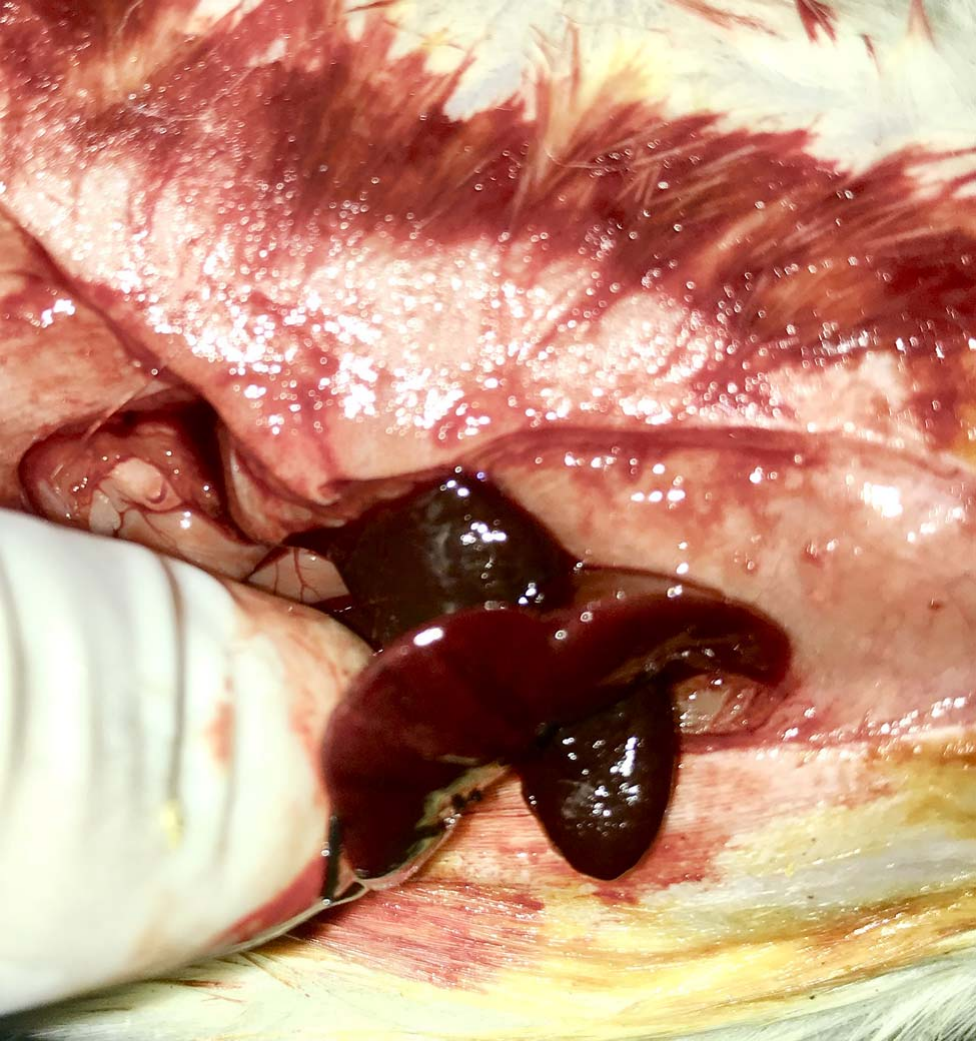
Patching liver laceration with autotransplantation of the spleen.

In the control group, a 2-cm length, full-thickness injury was made to the left lobe of the liver, and hepatorrhaphy was performed with the vertical mattress 3-0 silk sutures. A 1 cm×1 cm splenic fragments were cut and fixed with 3-0 silk sutures in the omentum. After completing hemostasis, the abdominal wall was closed. In the case group, instead of primary hepatorrhaphy, the 1 cm×1 cm splenic fragment was separated and fixed in the lacerated site of the left liver with a 3-0 silk suture (Fig. 1).

Finally, the incised skin region was repaired using a single layer of 3-0 nylon, and the rats were placed in an appropriate temperature (23–25°C) environment to become conscious. Following surgery, all animals were fed on a usual diet. The rats were monitored for 4 weeks after the surgery. On the seventh day, external sutures were removed under local anesthesia.

### Outcome assessment

Blood samples (10 ml) were collected by the capillary tube from the orbital sinus in the medial cantus 1 day before and 4 weeks after the operations in both groups. ELISA (monoband kit, USA) was used to measure complement proteins (C3 and C4), hemoglobin (Hb), immunoglobulins M, A, and G (IgM, IgA, and IgG), and platelet (Plt) and white blood cell (WBC) count. The intervention’s potential risks were evaluated, such as those connected to the systemic anesthetic, infection, hemorrhage, and mortality.

Additionally, after 4 weeks, we administered Technetium 99m sulfur colloidal (Tc^99m^). We performed scintigraphy using the Adac Forte machine to evaluate the function of the splenic fragments in the omentum versus the liver parenchyma. The process began with an intravenous injection of one millicori Tc^99m^ into the rat’s tail vein; after 2 min, an anterior 128×128 matrix with a high-resolution collimator image was acquired. Following completion of the surgery, all spleens were removed, and an area of interest was drawn around the spleen using the Adac machine’s Pegasus software.

In the SAO group, after primary scintigraphy, to confirm uptake by the transplanted spleen fragments, after giving sedation, a midline laparotomy was performed. The implanted splenic graft was removed, and scintigraphy using the Adac Forte machine was taken from it.

### Statistical procedure

All analyses used the SPSS (version 18.0; IBM Corp., USA). Statistical significance was indicated by a *P* value less than 0.05. Numerical variables are reported as mean and SD and, where appropriate, median and interquartile range. The normality of the distribution was tested using the Shapiro–Wilk test. The body weight of rats before surgery, Technetium uptake after surgery, and mean difference of changes in numerical variables before-after surgery were compared using independent sample *t* test or Mann–Whitney *U* test between groups. Paired samples *t* test was used to investigate the changes in mean values of variables from before to after surgery in each study group.

## Results

Either preoperative or postoperative comparisons between the two treatments showed no statistical significance in the blood levels of Hb, Plt, WBC, IgA, IgM, IgG, C3, and C4 (all *P*>0.05). Moreover, both groups had no statistically significant difference in radioisotope uptake after intervention (*P*=0.3). Descriptive statistics of both groups are shown in Table 1.

**Table 1:** Variables of the study in each group before and after interventions

Variables	Patch on the liver		Spleen omental autotransplant	
	Mean±SD	Median (IQR)	Mean±SD	Median (IQR)
Hb (g/dl)				
Before	16.8±0.7 [16.3–17.2]	16.75 (1)	16±1.3 [15.1–16.6]	16.10 (2.4)
After	14.4±1 [13.8–15.0]	14.35 (1.7)	13.6±1.4 [12.1–14.3]	13.65 (2.4)
WBC (/mcl)				
Before	9566.7±1493.8 [8617.5–10 515.8]	9900 (2750)	9800±1753.4 [87 56.2–12 013.0]	9800 (2775)
After	9033.3±1710.0 [7946.8–10 119.8]	8900 (2825)	12 341.7±1776.3 [11 428.6–13 740.7]	13 050 (2950)
Plt (/mcl)				
Before	796 166.7±115 775.5 [722 606.4–869 726.9]	788 500 (184 000)	745 000±131 295.6 [676 318.8–835 527.4]	770 000 (187 250)
After	839 833.3±109 330 [770 368.3–909 298.3]	860 000 (192 500)	738 250±122 589 [676 612.5–824 772.1]	770 000 (179 250)
C3 (mg/dl)				
Before	83.2±10.7 [76.4–90]	84.50 (13)	90±7.5 [84.7–93.9]	89.00 (13)
After	70.7±9.9 [64.4–77]	68.00 (14)	71.3±9 [65.8–76.1]	69.50 (12)
C4 (mg/dl)				
Before	9.7±1.8 [8.6–10.9]	9.50 (2.8)	10.1±2.0 [8.4–11.1]	10.00 (2.8)
After	8.6±1.9 [7.4–9.8]	8.00 (3.8)	6.9±2 [5.9–8.3]	7.00 (3.8)
IgG (g/l)				
Before	220.3±19.6 [207.9–232.8]	216.50 (38)	215.3±19 [197.8–225.3]	220.00 (32)
After	205.7±20.8 [192.5–219]	206.00 (38)	201.2±23 [189.1–216.6]	210.00 (32)
IgM (g/l)				
Before	31.1±8 [26.0–36.1]	30.00 (10)	28.1±6.1 [24.4–31.8]	25 (10)
After	31.8±7.4 [27.1–36.5]	30.50 (6)	23.5±7.4 [19.7–29.7]	22.50 (12)
IgA (g/l)				
Before	4.5±1.1 [3.8–5.2]	4.7 (1.7)	5.1±0.4 [4.9–5.4]	5.1 (0.7)
After	3.2±1.5 [2.2–4.2]	3.35 (2.8)	2.14±0.91 [2.1–3.2]	2.85 (1.4)
Radioisotopes uptake	1293.3±146 [1200.6–1386.1]	1310.00 (175)	1216.9±227.9 [1086.5–1350.3]	1156.50 (422)

C3, 4, complement protein 3, 4; Hb, hemoglobin; IgA, IgG, IgM, immunoglobulin A, G, M; IQR, interquartile range; Plt, platelets; WBC, white blood cell.

**Figure 2:**
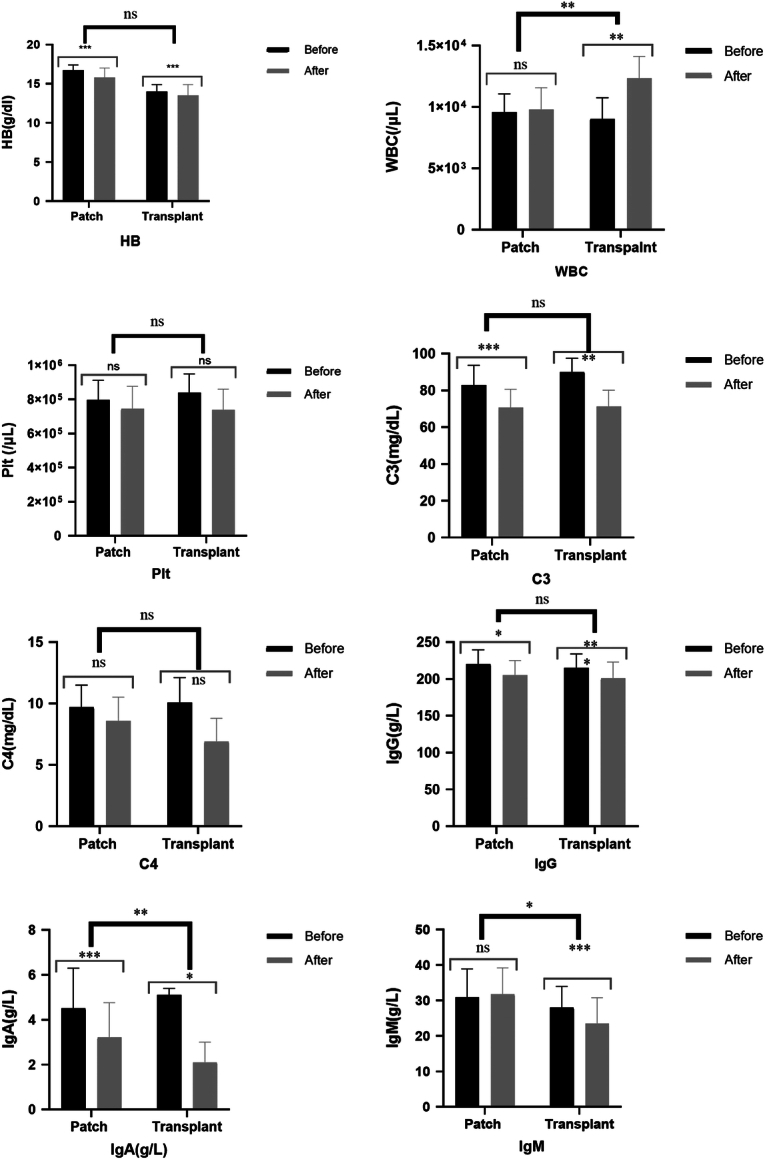
Comparison of preoperative and postoperative hematologic and immunologic variables in each group [Patch=spleen autotransplantation on the liver, SAL group (*n*=12). Transplant=spleen autotransplantation in the omentum, SAO group (*n*=12)].

Both groups significantly decreased Hb, C3, IgG, and IgA levels after spleen transplantation (Table 1 and Fig. 2). In the SAO group, WBC counts significantly increased but did not significantly change in the SAL group. On the other hand, postspleen transplantation IgM levels significantly decreased in the SAO group, while they almost did not change in the SAL group. Moreover, posttransplantation levels of Plt and C4 remain unchanged in both groups.

The mean difference of the variables (before surgery) was also compared between the two groups (Fig. 2). The mean differences of WBC, IgM, and IgA were significantly different between the two groups; the increase in WBC was higher in the SAO group, and for IgM and IgA, the decrease was more profound in the SAO group. For other factors, the difference was not statistically significant.

## Discussion

Both of the spleen preserving techniques for dual traumas of abdominal solid organs were the same in bringing maintenance of spleen function to the system based on the radioactivity detection on scintigraphy and finding a nonsignificant difference between changes in some of the hematological (i.e., Hb and Plt) and immunological factors (i.e., C3, C4, and IgG). However, regardless of the technique, separation and exclusion of the spleen from regular physiologic sites significantly affect the body’s hematological and immunological factors determined by the decrease in the Hb, C3, IgG, and IgA levels in both groups. The superiorities of the SAL technique are escaping from omental transplant potential complications (e.g., obstruction, anemia, necrosis, and abscesses) despite none seen in our study but has been found in others’ reports^6^. The SAL technique enables simultaneous treatment of both hepatic and splenic injuries and the continuation of the splenic function through the proposed procedure, such as patching the liver with fragments of the spleen obtained from splenectomy. By using SAL, there is no need to use local surgical hemostatic equipment like absorbable hemostat agents and balloon tamponed techniques to control bleeding in the significant and deep liver laceration, which could potentially induce infection. Finally, the SAL technique results in a lower decrease in IgA and IgM levels after splenectomy, two of the body’s most critical immunoglobulins.

The WBC level increased significantly in the SAO group, and the change was more noticeable than in the SAL group. Conversely, a similar experimental study revealed no difference in postintervention WBC levels between omentum and liver patch groups^8^. According to another study^14^, WBC level was temporarily elevated after splenic artery embolization due to increased splenic vascularization but returned to normal levels after 40 days. In contrast, WBC levels remain enduringly high in patients who undergo splenectomy^15^. Given that reactive increases in WBC levels were observed after splenectomy, the absence of a significant increase in WBC levels in the SAL group may imply greater activity of the splenic tissue in this group compared to the SAO group.

A significant decrease in the level of blood complement C3 suggests a decrease in splenic activity. Nevertheless, given their maintenance in mean different levels (C3 and C4), this may indicate that there was partial preservation of the spleen’s immunological activity, which scintigraphy supports. Similarly, another study showed no differences in C3 levels between liver patch and omental transplant groups^11^. Asplenic patients cannot activate the complement through the alternative pathway. This makes them more vulnerable to severe infections that can progress rapidly and become life-threatening^12^. Our study supports previous research indicating splenic autotransplantation can restore normal splenic filtration function and immunoglobulin levels^14^.

IgG level decreased in both groups; however, this change was not significant between the two groups. This suggests that both methods are equally effective in maintaining an appropriate level of IgG. Similarly, a study on rabbits found no differences in IgG levels between groups where splenic tissues were transplanted into the liver and omentum^11^. IgA levels were decreased in both groups, which was in line with another study^12^. Conversely, Karahan*et al*.^11^ showed that the levels of IgA do not change afterward. The participants in Ahluwalia and colleagues study were human, which can explain the controversial outcomes.

Furthermore, the mean difference of IgA was different between the two groups, and it favored the SAL group. This difference shows that splenic tissues in the liver may preserve the immune system better than the other group. For IgM, the results were inconsistent. In the SAO group, it was significantly decreased; however, it did not change significantly in the SAL group.

Moreover, the mean difference of IgM was different between the two groups, which favored the SAL group. The better function of the marginal zone of transplanted splenic tissues in the SAL group could explain this discrepancy in IgM levels. Conversely, Badawy *et al*.’s^8^ study on rabbits revealed no significant difference in terms of IgM between liver and omentum groups. The difference between Badawy and colleagues and our findings and our findings may relate to the included animal for the experiment, indicating that this area of spleen-preserving techniques should be tested with limited human participants to interpret the results better.

### Limitations

To determine the limitations of this study, the outcomes are based on the limited sample of rats, and animal results are not necessarily applicable to humans. However, the findings of this study are promising. Second, a more extended follow-up period is required to assess the function of transplanted splenic tissue on the liver. Ultimately, similar research has to be conducted on more giant animals (such as dogs and pigs) and those with similar immunologic systems before being applied to humans. However, as a case-report study on humans, we believe in the simultaneous injury of the liver and spleen that splenectomy and hepatorrhaphy are inevitable; the findings of this study could be executed.

## Conclusion

Autotransplanting splenic tissues as a patch in the lacerated liver parenchyma maintains splenic function similar to SAO. Considering better immunological functions, patching liver laceration with fragments of the spleen may be a valuable procedure to preserve splenic function in the simultaneous trauma of the liver and spleen without the need to perform the use of absorbable hemostatic agents or insert a tampon or a balloon to control the hepatic parenchymal bleeding.

## Ethical consideration

The present study followed national and institutional guidelines for animal treatment and complied with relevant legislation. The present study involved client-owned animals, demonstrated a high standard (best practice) of veterinary care, and involved informed client consent. The study has been approved by the University Ethics Committee according to code IR.MUI.MED.REC.1397.089 and has been performed in accordance with the ethical standards presented in animal treatment welfare.

## Consent for publication

None.

## Source of funding

This experimental study was not funded in any way.

## Conflicts of interest disclosure

There are no conflicts of interest.

## Research registration unique identifying number (UIN)

None.

## Guarantor

None.

## Availability of data and materials

The datasets used and analyzed during the current study are available from the corresponding author through the e-mail address on reasonable request.

## Provenance and peer review

Not commissioned, externally peer-reviewed.
